# Associations of physical strength with facial shape in an African pastoralist society, the Maasai of Northern Tanzania

**DOI:** 10.1371/journal.pone.0197738

**Published:** 2018-05-31

**Authors:** Marina L. Butovskaya, Sonja Windhager, Dimitri Karelin, Anna Mezentseva, Katrin Schaefer, Bernhard Fink

**Affiliations:** 1 Institute of Ethnology and Anthropology, Russian Academy of Sciences, Moscow, Russian Federation; 2 National Research University, Higher School of Economics, Moscow, Russian Federation; 3 Social Anthropology Research and Education Center, Russian State University for Humanities, Moscow, Russian Federation; 4 Department of Theoretical Biology, University of Vienna, Vienna, Austria; 5 Moscow State University, Moscow, Russian Federation; 6 Institute of Geography, Russian Academy of Sciences, Moscow, Russian Federation; 7 Department of Evolutionary Anthropology, University of Vienna, Vienna, Austria; 8 Department of Behavioral Ecology, University of Goettingen, Goettingen, Germany; 9 Leibniz ScienceCampus Primate Cognition, Goettingen, Germany; 10 Hanse-Wissenschaftskolleg, Institute for Advanced Study, Delmenhorst, Germany; SUNY Polytechnic Institute, UNITED STATES

## Abstract

**Objectives:**

Previous research has documented associations of physical strength and facial morphology predominantly in men of Western societies. Faces of strong men tend to be more robust, are rounder and have a prominent jawline compared with faces of weak men. Here, we investigate whether the morphometric patterns of strength-face relationships reported for members of industrialized societies can also be found in members of an African pastoralist society, the Maasai of Northern Tanzania.

**Materials and methods:**

Handgrip strength (HGS) measures and facial photographs were collected from a sample of 185 men and 120 women of the Maasai in the Ngorongoro Conservation Area. In young-adults (20–29 years; *n* = 95) and mid-adults (30–50 years; *n* = 114), we digitized 71 somatometric landmarks and semilandmarks to capture variation in facial morphology and performed shape regressions of landmark coordinates upon HGS. Results were visualized in the form of thin-plate plate spline deformation grids and geometric morphometric morphs.

**Results:**

Individuals with higher HGS tended to have wider faces with a lower and broader forehead, a wider distance between the medial canthi of the eyes, a wider nose, fuller lips, and a larger, squarer lower facial outline compared with weaker individuals of the same age-sex group. In mid-adult men, these associations were weaker than in the other age-sex groups.

**Discussion:**

We conclude that the patterns of HGS relationships with face shape in the Maasai are similar to those reported from related investigations in samples of industrialized societies. We discuss differences between the present and related studies with regard to knowledge about the causes for age- and sex-related facial shape variation and physical strength associations.

## Introduction

Physical strength and strength performance capacity are sexually dimorphic [1–2]. Men are typically stronger than women due to higher amounts of muscle mass, especially in the upper body [3–4]. Muscular strength correlates with a range of health outcomes, such as physical functioning [5] and cardiorespiratory fitness [6], and higher all-cause mortality rates and morbidity [7–8].

One commonly used measure of physical strength is handgrip strength (HGS). Handgrip strength is simple to administer, correlates with strength in other muscle groups, and displays a robust sexual dimorphism [9–11]. Isen et al. [12] reported that the additive genetic variance of HGS is higher in men than in women, suggesting that male strength shows greater phenotypic variance due to sex-related genetic expression, which renders men more susceptible to androgenic effects on HGS development. This could explain why in men more than in women, testosterone (T) has potent effects on the musculoskeletal system by increasing lean body mass and muscular strength [13–14]. Studies investigating effects of T supplementation have indeed documented a significant impact upon muscle size and strength and on body fat distribution [15–17].

In many species, including humans, the production and metabolism of T mobilize resources that encourage males to attract and compete for mates [18–19]. Testosterone affects sexually dimorphic facial morphology [20–21]. In pubertal males, high T levels facilitate the lateral growth of the cheekbones, mandibles, and chin, the forward growth of the bones of the eyebrow ridges and the lengthening of the lower face leading to a more robust face shape [22–23]. The influence of estrogen (E) leads to a more gracile facial shape with high eyebrows, less robust jaws, and fuller lips. Previous studies reported positive relationships of male facial masculinity with perceived health [24] and fertility [25], which may explain why women often report a preference for masculine looking male faces [26], especially in short term-context and when they are fertile [27]. However, it is also likely that the female preference for certain male faces arise from the fact that dominant- and masculine-looking males are signaling characteristics which may be beneficial in intra-sexual conflict [28], and thereby also indicate potential achievers of high status. Behavior which intends to achieve, maintain, and enhance status is observed primarily amongst high T individuals [29]. If certain facial configurations indeed relate to potential for high status in males, then, cues that reflect this quality may be used by both sexes in contexts of inter- and intrasexual selection [30–32].

Investigations of people’s perceptions of male facial and body morphology have shown that both men and women can accurately assess male physical strength from facial and body images and use this information to evaluate male fighting ability [33]. Moreover, Fink et al. reported that women rated faces of physically strong men (assessed via HGS) as attractive, dominant and masculine [26]. Windhager et al. employed the geometric morphometric toolkit (GMM) to statistically assess and visualize the covariation of facial shape and HGS (among other anthropometric measures) and (female) perceptions of dominance, masculinity and attractiveness in a sample of German men [34]. Physically strong men could be characterized by robust-looking, round faces, wide eyebrows and a prominent jaw outline. Facial configurations associated with physical strength were similar but not equal to those linked to assessments of dominance and masculinity. Face shape associated with attractiveness ratings somewhat resembled facial configurations that related to body height. Thus, conclusions about relationships of facial morphology with perception data, should be treated with caution when considering correlational data alone [35].

It has been unclear whether the reported associations of physical strength and face shape extend to other (non-industrialized) societies. In the present study, we investigated associations of face shape and HGS in a small-scale society, the Maasai of the Ngorongoro Conservation Area in Northern Tanzania. Previous studies suggest that physical strength signifies fighting ability, and thus resource holding potential [33,36], which is crucial in sexual selection, especially in contest competition [37]. Considering previous findings from investigations of physical strength assessments based on facial and body photographs in horticulturalist and pastoralist societies [33], we expected to identify significant relationships of face shape and physical strength in the semi-nomadic, pastoralist Maasai. Given the reported effects of T on the development of physical strength, especially in males, we hypothesized that facial configurations associated with strength in the Maasai would be similar to those previously reported in Western samples.

## Materials and methods

### The Maasai culture

The Maasai are the Maa-speaking pastoral people of Tanzania. According to a census conducted in 2007 their population in the Ngorongoro Conservation Area (NCA) was 70,000 [38]. The Maasai culture and social structures are highly conserved but also adaptable to changes [39]. About half of the married men are in polygynous relationships with an average of 2.8 wives per person [40]. A system of territorial groups (sections), clans and age-sets provide the basis of social and economic cooperation. Currently, the Maasai of the NCA are exclusively pastoralists, as any sort of agricultural activity is not allowed inside the habitat [41]. Each section has its own land, and only members of this section are allowed to graze their stock within this territory [42]. Sections are divided into localities and within a locality grazing land is controlled by neighborhood “bomas” (“households” in Swahili). Particular families may have priority rights over local water resources. Clan relationships are inherited from the father and lineages are usually three generations deep. There are seven clans [42] and up to 100 subclans [39]. Marriages are often arranged between members of different clans, but if a clan becomes very large, then intermarriage may happen between subclans [43]. Members of individual clans are dispersed across different sections.

The age-set system plays an important role in men’s life. Adjacent age-sets are in lifelong political and ritual opposition and competition. Every 15 years, a new age-set is opened. All boys of suitable age become circumcised during this period and join a group of “il murran” (“junior warriors”). Traditionally, the new murran establish a “manyatta” (“warrior camp”) to protect their locality and organize a ritualized raid on their parental bomas to take away married women (their mothers) and cattle from fathers’ herds. Mothers build huts in the manyatta and provide a home base for the murran. Murran spend time with travelling, herding, and feasting in the bush. They are visiting elders’ bomas, where elders usually treat them with both tolerance and suspicion due to the power the murran have gained in their new social role [39]. Murran also tend to visit young, unmarried girls for whom they can be protectors, friends and lovers. However, despite strong attachment of such couples, girls are usually given by their fathers to men, who have finished the period of murran life. In many cases friendship between former murran and girls are very strong, and mutual reciprocal aid between men and women remains lifelong [43]. The older age-sets usually control the younger.

### Data collection and participants

Our data were collected in January and June 2016 among a Maasai population settled around Endulen village, located in the NCA, as part of a long-term project run by one of us (MLB), which investigates the biological and cultural characteristics of pre-industrial societies in Tanzania. Members of this population are traditional pastoralists, i.e., they do not practice any cultivation, live in typical Maasai bomas and consume traditional Maasai food (milk, meat and blood). Maize and beans are added to the diet, and these resources are partly provided by NCA administration as compensation for prohibition of cultivation in this area. They have limited contacts with people outside the area, including tourists, because tourists are mainly traveling to the Ngorongoro Crater or to Serengeti. The Maasai group in this area follows traditional rules on initiations, murran practices and (to some extent) traditional marriage patterns [41].

Our initial sample was 305 individuals (185 men, 120 women). They reported ages from 17 to 90 years. In the present study, we considered two types of information, i.e., HGS data and facial images, to investigate possible associations of the two by assessing facial morphology through the application of geometric morphometric analysis. Four participants were excluded from analysis because of missing HGS data. Focusing on HGS and face shape in young adults and mid-adults further reduced the sample for tests of relationship between these variables to 120 men and 89 women.

### Ethics

Ethical approval of the study protocol and consent procedures was obtained from the ethical committee at Moscow State University (protocol #55, 2015) and the Tanzania Commission for Science and Technology (COSTECH) provided research permission protocols (#2015-117-ER-2009-151 and #2017-185-NA-2009-151) Informed consent was obtained from all participants, either written or verbally in case a participant was not literate.

### Anthropometric measures

**Fig 1:**
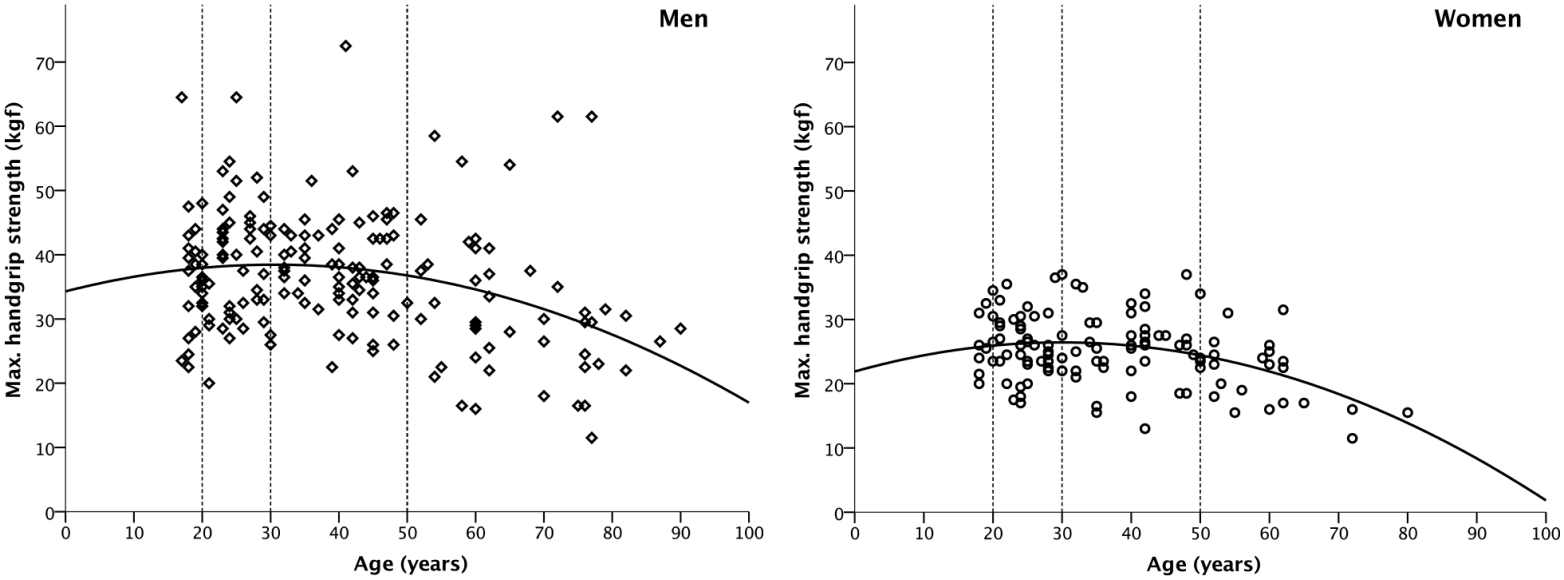
Scatterplot of handgrip strength as a function of age, separately for Maasai men (left) and women (right). Cut-off points for the two age groups included in the shape analysis are indicated with vertical, dashed lines. The curved line is the quadratic regression of physical strength on chronological age (men: *R*^2^ = 0.10, *n* = 183; women: *R*^2^ = 0.14; *n* = 118).

Handgrip strength (in kgf) was assessed with a portable hand dynamometer (DMER-120, Tulinovsky Instruments, Tulinokva, Russia). Participants were instructed to squeeze the dynamometer as hard as they can, in standing position, and with the arm stretched downwards. Left and right HGS was measured twice and the highest of the four measurements was used in the statistical analyses. Handgrip strength is associated with age [8], and this was also found in our sample (see Fig 1). We therefore focused on the following two age groups in the investigation of HGS and facial shape associations: young adults (20–29 years, *n* = 95) and mid-adults (30–50 years, *n* = 114). Within these age groups, HGS and age were not significantly correlated (Table 1).

**Table 1:** Correlations (Spearman *r*_*s*_) of handgrip strength with age within age-sex groups.

Sex	Age group (years)	*r*_*s*_	*p*	*n*
Male	< 20	.063	.817	16
	20–29	.191	.166	54
	30–50	−.067	.595	66
	> 50	−.266	.070	47
Female	< 20	.474	.282	7
	20–29	−.118	.462	41
	30–50	.048	.746	48
	> 50	−.459	.032	22

*p*-values are two-tailed and not corrected for multiple comparisons.

### Facial photographs

Participants were seated in approximately 1.80 m distance to the camera and instructed to look straight into the lens (Nikon D90, 70 mm lens equivalent to 105 mm for 35 mm film) while maintaining a neutral facial expression. A scale bar (in cm) was included in each image. Faces were positioned visually according to the Frankfort Horizontal Plane (FH) with the lens at eye height.

### Morphometric analysis

Seventy-one landmarks and semilandmarks were digitized on each face following Windhager et al. [34] and by adding “vertex” (highest point of the head when oriented according to the FH [44]). These landmark configurations were adjusted to the scale bar, subjected to a Generalized Procrustes superimposition [45], and symmetrized [46–47], separately for each age-sex group. The resulting shape coordinates were then regressed upon HGS. The strength of this association between HGS and facial shape was quantified by correlation of shape scores with HGS (see [48] for the procedure). Because we did not expect the standard linear regression of shape onto HGS to “explain” a large part of the variation [34], we projected the shape data (vectors) for each individual orthogonally onto the regression line and used the correlation between these scores and the original HGS scores to assess the meaningfulness or strength of the observed relationship. Permutation tests [49] with 10,000 permutations were used to test for its statistical significance. The corresponding facial shape patterns were visualized in the form of thin-plate spline (TPS) deformation grids [45], superimposed by cubic splines between landmark points to facilitate interpretation, as well as by image unwarping and averaging (“geometric morphometric morphs” [50]).

### Software

We adopted *tpsDig2* 2.17 for landmark digitization, *tpsRelw* 1.67 for sliding of the semilandmarks and *tpssuper* 2.04 for the image unwarping and averaging [51]. Mathematica 9 was used for symmetrization, shape regressions and permutation tests. SPSS 23 was used to estimate the association between physical strength and age.

## Results

**Fig 2:**
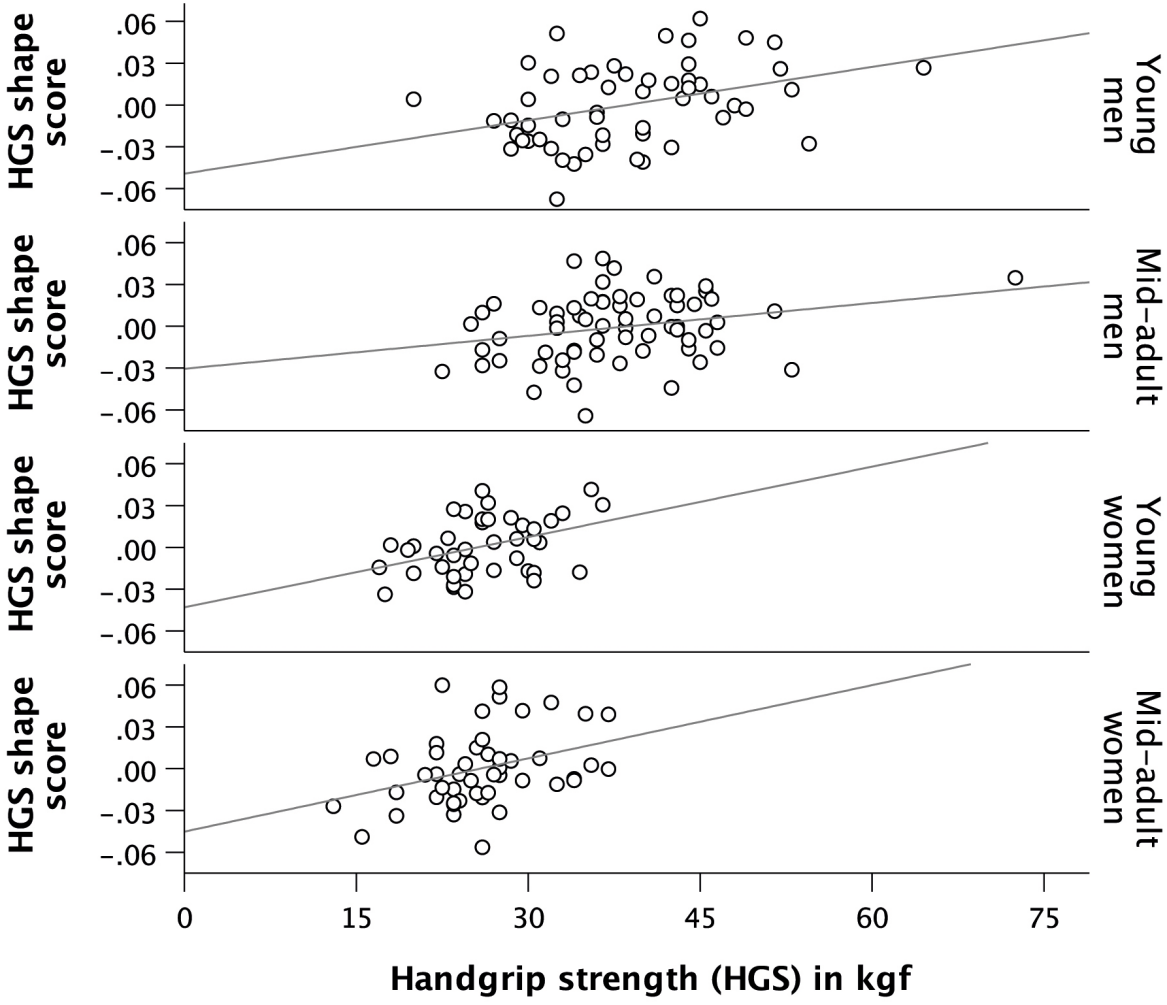
Visualization of the association of handgrip strength (HGS) with the corresponding facial shape scores within age-sex groups. The test statistics related to the depicted regression lines are given in Table 2. The association is significant in all sex-age groups, but stronger for women and young men than for mid-adult men.

**Fig 3:**
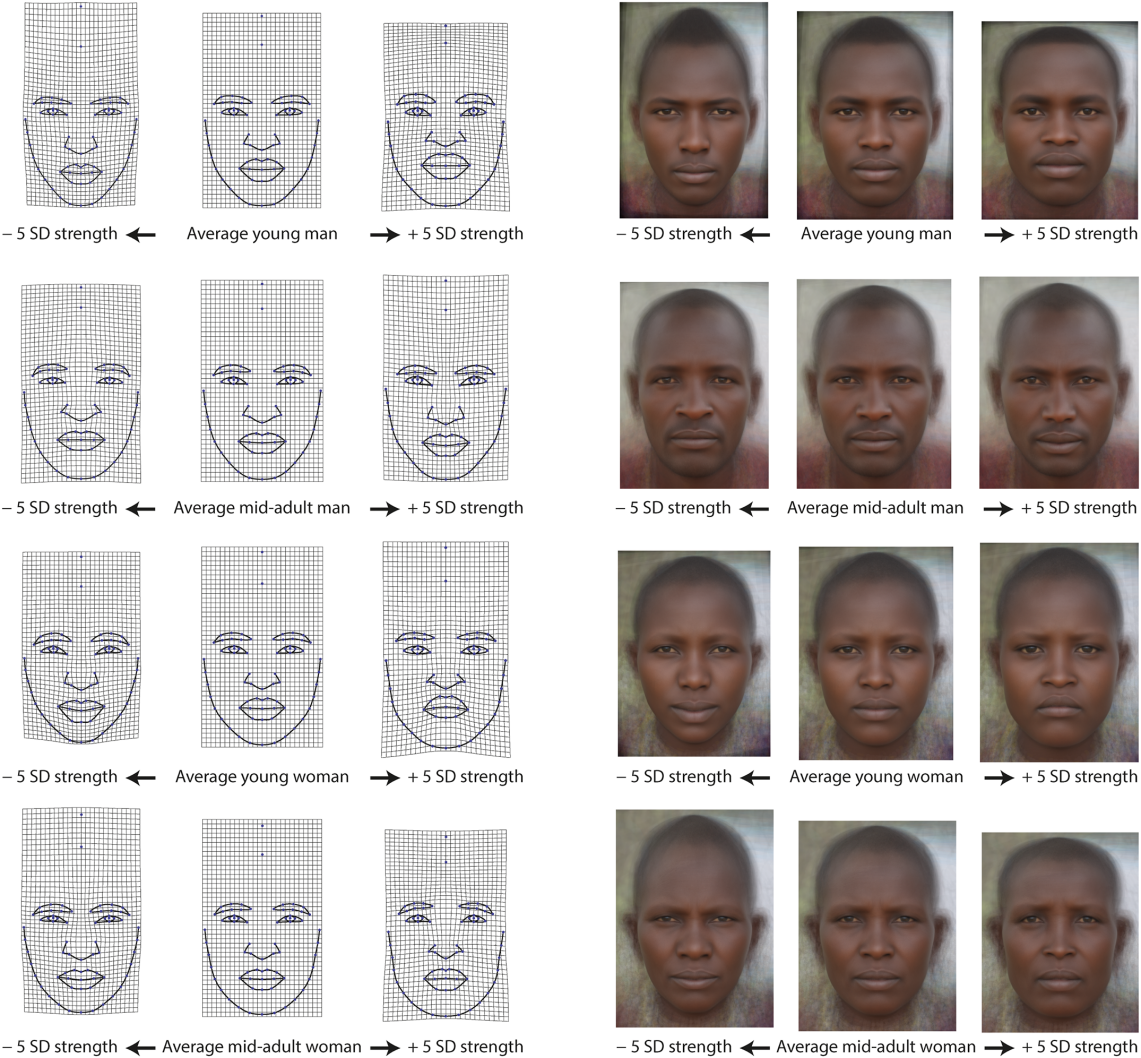
Facial shape correlates of handgrip strength (HGS) in the Maasai. Thin-plate spline deformation grids (on the left) depict the facial shape changes with HGS as deformations from the average facial configuration (middle column) to −5 SD of HGS (left column) and to +5 SD (right column), separately for each sex-age group. This magnification factor was applied to facilitate interpretation. The same facial configurations were also visualized through image unwarping and averaging (on the right). Thin-plate spline deformation grids and facial morphs were aligned at the height of the pupils and scaled for interpupillary distance.

Facial shape and handgrip strength were significantly associated in all four age-sex groups (Table 2, Fig 2). Following the nomenclature of Cohen [52], a medium sized effect was found for young men and young women, and mid-adult women with correlation coefficients larger than .35. In mid-adult men, the effect size was smaller (*r* = .26), and the shape pattern less consistent in comparison with the other three groups (Fig 3). In male and female young adults and mid-adult women, Maasai with higher HGS tended to have wider faces with a lower and broader forehead, a wider distance between the medial canthi of the eyes, a wider nose, fuller lips, and a larger, squarer lower facial outline compared with weaker individuals of the same age-sex group (Fig 3). In contrast, faces of relatively weak individuals were more elongated and gracile, the distance between the medial canthi of the eyes as well as the visible part of the iris and sclera were smaller, the nose narrower, and the chin more pointed. The lower face was relatively small compared to that of stronger individuals and to the upper part of the face. These oblong faces had mouths with upturned corners (despite a neutral facial expression). In young men, the shape regression further showed a difference in relative brow size with stronger men having thicker eyebrows (Fig 3, upper row). This feature was also present in mid-adult men as was the increase in lip fullness and relative eye size with increasing HGS. The relative size and shape of the lower face, however, indicated a broader jowl and chin area in weaker individuals.

**Table 2:** Test statistics for the association of handgrip strength with facial shape scores within age-sex groups.

	Shape scores		
	*r*	*p*	*n*
Young men	0.370	0.006	54
Mid-adult men	0.261	0.034	66
Young women	0.392	0.011	41
Mid-adult women	0.354	0.014	48

All *p*-values are the result of permutation tests based on 10,000 permutations.

## Discussion

We investigated the association of facial shape and HGS in men and women of the Maasai and found evidence for a relationship similar to previous reports in Western samples (for German men [34], for UK men and women [53]). Windhager et al. reported in a sample of young German men (18–31 years) that faces of stronger men tended to be more robust [34]. They had a wider facial outline, a pronounced lower face, and wider eyebrows compared to faces of weaker men. Given overall similarities of facial morphology relationships and HGS in the Maasai with that reported from a Western sample, we propose that the patterns of associations are genuine and applicable across populations and societies. However, the strength of the face–strength relationship may show considerable variation depending on socio-cultural organization, and thus the significance of physical strength in a society. In the case of the Maasai, this might explain why the relationship of facial shape and HGS in mid-adult men was less coherent.

Physical strength is particularly important for young (murran) men. Traditionally these men herd cattle and protect them from carnivores. They also act as warriors, defend local households from raids of neighboring tribes (Datoga and Sukuma) and in turn steal cattle from them [41]. Those who are more successful in these activities gain higher social prestige, and this may facilitate access to female mating partners. Murran men, therefore, frequently practice spear throwing, bow shooting, and fighting with sticks. Competition is particularly strong among members of this group, as the display and practice of skills associated with physical strength offers considerable benefits. Around the age of 30–35 years, a man’s social role changes. Men are no longer murran but are allowed to stay with their wives and to eat together with them. Physical skills and competition do no longer play a prominent role and social status is at that time reflected by the number of cattle they own, as well as their social skills. We therefore speculate that the weaker relationship of HGS with facial morphology in mid-adult men is–at least in part–the result of the Maasai age-set system, which puts particular selection pressure on young men to develop and maintain physical strength. Thereafter, heterogeneous living conditions and lifestyles resulting from differential wealth might obscure the face–strength relationship in that these effects increase facial shape variation for any given amount of HGS.

It has to be noted that the majority of related investigations in samples of industrialized societies considered individuals of ages 20–30 years. Thus, comparing previous reports from Western samples with our present data means that we are considering male Maasai, for which selection pressure due to male competition might be stronger than the typical forces acting upon a young man in his 20ies of an industrialized society. Moreover, there is little reason for the hypothesis that the face-strength relationship would not become weaker also in Western samples. However, there might be differences in regard to the speed of the decrease of this relationship, with a more gradual decline (and at a slower pace) in Western societies. Yet, this is highly speculative given the absence of face shape and HGS information from older subjects that could be used for investigating similarities and/or differences with the patterns detected in the Maasai. Future studies investigating correlates of facial morphology should, therefore, extend the age range of their samples as this would offer the possibility to investigate age-related changes in the strengths of associations between facial form and its biological causes and consequences, including measures of reproduction and resources. An ideal scenario includes the collection of longitudinal data, thus tracking morphological changes and depending measures over a longer period of time in order to arrive at stronger conclusions about possible mediators of evolutionary and social mechanisms. In the present study, we sought to test direct relationships of HGS with facial morphology. Further investigations on the relative importance of face shape and HGS, especially for male and female assessments of mate qualities in the Maasai, would benefit from collecting information on resources, which are known to contribute to social prestige (e.g., number of cattle, [41]).

Our data also show an association between HGS and facial shape in young and mid-adult women. In contrast to men, obtaining and maintaining physical strength is important in Maasai women throughout their lives. First, there is strong competition among Maasai women who marry at the age of about 18 years, and then compete with co-wives for resources. Physical strength in women may therefore be regarded as quality trait, which indicates overall health including the physical capacity to carry heavier items (e.g., water from the river). Women, unlike men, in the Maasai are prescribed to conduct various household duties for the whole lifespan, and being physically strong facilitates these activities.

Taken together, the results of the present study corroborate the proposition of an association between physical strength (as measured by HGS) and facial shape. Our analyses detected significant correlations between HGS and facial shape in each of the four age-sex groups tested. Previous studies have reported associations predominately in male samples from industrialized societies. It has been proposed that effects of prenatal androgenization may account for part of the variance in male and female facial morphology [21,54]. In this view, high levels of prenatal T (as indexed via digit ratio, or 2D:4D) lead to the development of robust faces, and a prominent facial outline, whereas low levels of prenatal T renders faces more gracile in their appearance. This effect basically applies to both sexes and is independent from the effect of chromosomal gender. Related studies have documented 2D:4D relationships with HGS in men [55], and 2D:4D and dominance relationships [56]. Moreover, it has been demonstrated that the activity of the androgen receptor gene polymorphism (accounting for part of prenatal androgenization) in men from traditional African populations correlates with both aggression as well as the number of offspring [57–58].

It is likely that prenatal androgenization effects contribute to both facial morphology and physical strength, thus mediating the observation of face shape and strength associations. If true, one would hypothesize links in pre-industrialized societies similar to the reports from Western samples. The present data on facial shape and HGS relationships reports shape patterns in the Maasai, which are comparable to those in industrialized societies; however, we should be cautious with direct comparisons of existing studies, because none of the studies in industrialized societies has considered a larger age range in the assessment of face shape and strength associations. The conclusions were primarily derived from the investigation of young individuals, comparable to the age range of the young cohort in the present study. Thus, according to our knowledge, this study is the first to demonstrate that the facial shape association with HGS generalizes to older age.

Isen et al. reported that in men androgen-mediated mechanisms of physical strength are more strongly influenced by genetics, whereas in women environmental effects seem to play a stronger role [12]. Given the demands on Maasai women in daily life, in addition to the continuing challenges from female competition, it is perhaps not surprising that we detected significant associations of HGS with face shape not only in young but also in older women. Whether these associations can be found also in women of industrialized societies needs to be tested. Research suggests that in consequence of intra- and intersexual selection, pressure on young men is particularly high; thus, young men may develop and display male-typical features and behavior more pronounced than older men in order to outcompete others for the access of mating partners. Unlike in the Maasai, young men in industrialized societies are not assigned a “warrior” role (even though they may sometimes feel so), and the transition from “young” to “mid adult” is smoother. We speculate that this will be evident in facial shape and strength relationships. Clearly, we hypothesize that the relative strength of challenges from environment and socio-cultural organization across societies, including variation in mating systems, may find their expression also in differential patterns of face shape and strength relationships of both men and women.

There are some caveats with relating the present result on HGS and facial shape relationships to that of previous studies. Few studies have concentrated on direct relationships of physical strength measures and facial morphology, and sometimes numerical information is missing in previous reports. For example, Holzleitner and Perrett reported associations of HGS with facial shape in young British men and women [53]. Yet, they did not provide information about the range of HGS measurements in their sample and about the strength of relationship between HGS and facial shape. In addition, the shape vector was based on subsampling instead of regressing HGS on facial shape information of the whole sample. We consider it essential having access to this information when comparing findings across studies and populations, especially for conclusions about differential selection pressures which may account for part of the variation in facial morphology across societies. Such information has often not been provided in studies that focused on associations of facial appearance with perceived strength. When perceived and actual strength are compared, effect sizes have been reported to be small [33,53], thus rendering perceived strength an unreliable proxy for physical strength.

In conclusion, the results of the present study corroborate the assertion of an association between physical strength and facial morphology. The patterns of HGS relationships with face shape in the Maasai were similar to those reported in previous studies for samples of industrialized societies. This suggests that physical strength associations with face shape might be universal, even though local variation with regard to the strength of the relationship due to environmental and societal influences is plausible. Given the small number of studies that have tested direct relationships of physical strength measures with facial morphology, this conclusion remains speculative and needs to be substantiated by further investigation.
